# Effects of different stocking densities on performance and activity of cattle × yak hybrids along a transhumance route in the Eastern Himalaya

**DOI:** 10.1186/s40064-015-1175-4

**Published:** 2015-08-07

**Authors:** Shanker R Barsila, Naba R Devkota, Michael Kreuzer, Svenja Marquardt

**Affiliations:** ETH Zurich, Institute of Agricultural Sciences, Universitätstrasse 2, 8092 Zurich, Switzerland; Agriculture and Forestry University, Directorate of Research and Extension, Chitwan, Nepal

**Keywords:** Grasslands, Milk composition, Himalaya, Yaks, Hybrids, Nepal

## Abstract

Twelve lactating cattle × yak hybrids (*B. taurus* × *B. grunniens*) were investigated on five experimental pastures (Sites 1–5), following an up- and downward transhumance route (3,200, 4,000, 4,500, 4,000 and 2,600 m a.s.l.). Hybrids were kept in replicated groups of two (low SD) and four animals per paddock (high SD). As a control, yaks (with calves) were included at low SD at Sites 2–4. Performance was described by body weight, milk yield and composition. Herbage samples as selected by the animals were analyzed. Activity patterns of the hybrids were assessed at Sites 2–4. At similar body weight change and milk composition, the individual hybrids on average produced 26% less milk at high than at low SD. However, at high SD on average still 49% more milk/m^2^ of pasture area was produced. Milk fat increased with time from 5.4 to 7.1%, milk protein decreased from 4.2 to 3.3%. At high SD, the hybrids spent less time standing and more time lying. The yaks gained weight at 4,500 m a.s.l. while the hybrids lost weight (+836 and −653 g/animal/day, respectively). In conclusion, high SD reduced individual milk yield and forced hybrids to spend more time for feeding. The yaks apparently had an advantage over the hybrids at very high altitude.

## Background

F1-hybrids of yaks and cattle are common in the yak-keeping high altitude areas of Asia (Qi et al. 2009). In the Nepalese Himalaya, both yaks and hybrids are managed in traditional transhumant systems (Joshi 1982), where they are moved along with seasonal herbage growth across an altitudinal range (Dong et al. 2009). Hybrids have an advantage in milk yield (MY) due to heterosis (Wang et al. 1994). However, there are different types of hybrids that differ in their adaptability to high altitude grazing (Barsila et al. 2014). Milk and milk products of yaks and hybrids are the main sources of livelihood of the herders in these areas (Joshi 1982). However, rangeland productivity is declining due to overgrazing as has been reported for other mountainous areas (Mishra et al. 2001). In overgrazed rangelands, unpalatable shrubs cover increasing proportions of the area (Bauer 1990), which would further restrict the land available for livestock grazing and thus promote overgrazing.

Therefore, in the present experiment, two hypotheses were tested (1) if increased stocking densities will result in losses of individual animal performance and changes in the activity pattern of the animals and (2) if, at high altitudes, cattle × yak hybrids do no longer perform better than yaks because of the hybrids’ incomplete inheritance of the yak’s specific high altitude adaptive capacity. In order to be able to test the hypotheses, a controlled field experiment was conducted along a transhumant route starting from 3,200 m a.s.l. up to 4,500 m a.s.l. and descending to 2,600 m a.s.l., including five pasture sites used for data collection. The performance of the hybrids was compared with that of purebred yaks at the three high altitude pasture sites. The pasture site effect included altitude, season and sward composition (plant species, vegetative stage) as potential factors of influence.

## Methods

### Experimental sites and climate recordings

A traditional transhumant route used by herders of Olangchung Gola, Taplejung district, and five pasture sites (Sites 1–5 of 0.17, 0.30, 0.23, 0.21 and 0.14 ha, respectively) along this route, were selected for the experiment in 2010. The route is situated within the Kanchenjunga Conservation Area in the Eastern Himalayan Mountains of Nepal. In order to protect the areas from grazing, the pastures were fenced 2 weeks before the respective measurement periods started. All paddocks were relatively flat and had a North-East aspect. Sites 2 and 4 were established at the same pasture site; however, sites were not contiguous but separated by a small ridge. Sites 1–3 (at 3,200, 4,000 and 4,500 m a.s.l., respectively, upward movement) were grazed in spring and summer (May 19–27, July 6–14 and August 4–12 at Sites 1–3, respectively), while Sites 4 and 5 were used in late summer and autumn (4,000 and 2,600 m a.s.l., September 1–9 and October 30–November 7, respectively, downward movement). A portable weather station was installed at each of the five pasture sites. Average temperatures measured at 01.30 p.m. and minimum temperatures, recorded at 06.30 a.m. (both in °C) for Site 1 were 16 ± 5 and 4 ± 3, for Site 2: 14 ± 1 and 2 ± 1, for Site 3: 12 ± 1 and 2 ± 2, for Site 4: 12 ± 2 and 1 ± 1 and for Site 5: 11 ± 1 and 2 ± 1, respectively. Average precipitation (measured at 06.30 a.m., in mm/day) recorded was 7 ± 11, 9 ± 7, 12 ± 8, 4 ± 6 and 4 ± 5 for Sites 1–5, respectively.

### Experimental animals

All procedures involving the animals followed the international guiding principles listed by the Council for International Organizations of Medical Sciences and the International Council for Laboratory Animal Science (2012). Nepal had no established system for ethical approval of animal experiments. In the area, two different hybrids are common. The hybrids selected were female *Bos taurus* × *Bos grunniens* (♂ × ♀) hybrids, locally called Dimjo chauries, which were shown to tolerate higher altitudes better than the Urang chauries (*Bos grunniens* × *Bos indicus*) (Barsila et al. 2014). The hybrids used originated from Yangma, Olangchung Gola (4,200 m a.s.l.). In total, 12 female hybrids which had calved between April 7 and May 2 at 2,500–3,000 m a.s.l. were selected and allocated to two different stocking densities (SD), low and high. Animals of similar milk yield (MY) and body weight (BW) were chosen (average initial MY, May 14–16: 2.0 ± 0.20 and 2.1 ± 0.27 kg and initial BW, May 18: 201 ± 7.0 and 202 ± 12.2 kg in low and high SD, respectively). At Sites 2–4, high enough in altitude for yak husbandry, four female yaks were additionally included that had calved between May 15 and 20. The yaks had an average BW of 186 ± 3.9 kg and a MY (including the amount suckled by calves) of 3.3 ± 0.8 kg as measured on May 18 and July 4/5, respectively. In compliance with traditional practice, the yaks, but not the hybrids, were accompanied by their calves during the whole experiment.

### Experimental design, determination of forage biomass and paddock sizes

The hybrids were kept at two SD in fenced paddocks during measurement periods of 9 days per site each. The paddock sizes were calculated per site based on assessments of current forage biomass availability 2 days before starting the measurements and estimates of animal requirements considering actual BW and MY. Therefore, samples of the total biomass were harvested from 50 × 50 cm plots (Sites 1–5, *n* = 12, 3, 6, 6 and 4 plots, respectively). Only the green forage biomass was used for calculation of paddock size. It was separated into palatable biomass (i.e., forage) and unpalatable biomass (based on personal assessments of the herders). A dry matter (DM) content of 200 g/kg was assumed. Feed intake was estimated based on the animals’ requirements using the equation stated in Wiener et al. (2003) for lactating yaks:

$$ {\text{DM intake }}\left( {{\text{kg}}/{\text{day and animal}}} \right) \, = \, 0.008\,\times\,{\text{ BW }}\left( {{\text{kg}}^{0.52}} \right)\,+\,1.369\,\times\,{\text{MY }}\left( {{\text{kg}}/{\text{day and animal}}}\right). $$

The corresponding assessments for BW and MY were made one and two consecutive days before stocking the paddocks, respectively. Shortly before starting with the experimental periods, the final paddocks were established within the previously fenced areas. Additional space was allocated if part of the area was covered with unpalatable shrubs or stones. Estimates of trampling losses were considered. Based on these considerations, the amount of forage on offer was estimated to match the requirements of four adult hybrids at high SD during 9 days. Low SD was defined as stocking with only half of the animals for the same period of time in a paddock of similar size. At Sites 1–5, this resulted in paddock sizes (m) of 41 × 42, 55 × 54, 50 × 45, 50 × 42 and 40 × 35, respectively. The corresponding SD (m^2^/head) at Sites 1–5 was 861, 1,485, 1,125, 1,050, and 700 at low SD and 431, 743, 563, 525 and 350 at high SD, respectively. Both SD treatments were repeated by independent paddocks per site. Two additional paddocks, established at Sites 2–4, were stocked with two yaks per paddock (following the low SD scheme). The animals were moved into the paddocks after morning milking at about 09.00 a.m. on day 1 and were released at about 05.00 p.m. on day 9. Before starting the experimental periods, the animals had been kept for 5 days in the surroundings of the respective pasture site without entering the pre-fenced areas. In the time between the experimental periods, the animals were kept together with a larger herd of the respective genotype and were moved along the transhumance route according to the herder practice.

### Recordings and samplings made in the herbage

The total biomass availability was measured in three random plots of 50 × 50 cm/paddock 2 days before the animals were allocated to their respective paddocks. To measure biomass availability, samples were cut 2 cm above ground (Pande and Yamamoto 2006), resulting in total *n* = 12 and *n* = 18 plots at Sites 1 and 5 and at Sites 2–4, respectively. The standing biomass was separated by functional groups, first air dried and later dried at 60°C during 48 h in an oven, weighed and milled instantly in order to determine the amount of air DM. Samples of herbages as selected by the animals were collected per paddock from days 1–7 of the measurement period from 09.00 a.m. to 03.00 p.m. (and from 10.00 a.m. to 03.00 p.m. at Site 5). The animals were accompanied and their selection behavior was mimicked for the whole duration of the respective observation period by randomly switching between animals following the procedure described by Berry et al. (2002). Every 30 min sampling was resumed in another paddock. Per paddock, two independent samples (A and B) were collected. In the evening, the samples of 150–200 g fresh weight were pooled across sampling days by maintaining separate A and B samples, chopped to small pieces and spread within a tent to facilitate drying.

### Measurement of milk yield and composition

Hand milking was accomplished in the morning and the evening (the latter only in the hybrids). The MY was measured with a digital balance (Scout Pro, Ohaus, NJ, USA). In the yaks, at first milk let-down had to be initiated by allowing the calves to suckle for 1–2 min for two times, then hand-milking was practiced and finally the calves were allowed to suckle again. By weighing them before and after suckling, the milk amount consumed by the calves was determined. Keeping yaks and their calves separately at night ensured that most of the milk was consumed in the morning. Still, the actual total MY of the yaks was probably higher as the calves may have suckled also during the day. Milk compositional analysis was done in duplicate after each milking in the 9-day experimental periods by a portable ultrasonic milk analyzer (Lactoscan SA-L, Milkotronic Limited, Nova Zagora, Bulgaria). For the hybrids, aliquots (weighted means) of morning and evening milk yields were used for calculation of the daily values. The contents measured were adjusted using regression equations obtained from 25 milk samples analyzed with the portable milk analyzer and the standard Milkoscan 4000 (Foss Electric, Hillerød, Denmark) (for details see Barsila et al. 2014). For statistical analysis, the average of the last 3 days/experimental period was used. Energy corrected milk (ECM, kg/day and animal) was calculated as milk (kg/day and animal) × (0.38 × fat (%) + 0.24 × protein (%) + 0.17 × lactose (%)/3.14 (Agroscope 2015).

### Measurement of body weight

Body weight (BW) was assessed with a digital balance (BH-300X, Aditsan, New Delhi, India, accuracy of ±0.02 kg) covered by a wooden platform of a size of 200 × 50 × 5 cm. Calves were weighed before and after each milking. Adults were weighed after morning milking 1 day before the start of each experimental period and after evening milking on day 9. The daily BW change was calculated as (initial BW minus final BW)/9 days.

### Recording of the activity pattern in the hybrids

Times spent walking, standing and lying, and the number of steps were recorded on Sites 2–4 in always the same two hybrids per paddock (*n* = 8) by accelerometer-based sensors to record animal behavior (IceTag 3D, IceRobotics Ltd., Edinburgh, UK). The sensors were fixed at the right hind leg in the morning of day 1 of the experimental periods during milking and detached when the animals were released at the evening of day 9. Thus, complete data sets were only available for 8 days. Data was evaluated by the corresponding software (IceTag Analyser 2008, IceRobotics Ltd., UK). Analysis was done per minute following Aharoni et al. (2009) by considering the activity as either standing or lying according to the highest proportion (i.e. >50%/min). Standing was further separated into walking (>3 steps/min) and standing (≤3 steps/min). To account for the times needed for gathering of the animals and for milking both in the morning and evening, a total of 122 min was removed from the dataset of each daily record, resulting in 539 min of time allocated to daytime (09.00 a.m. to 05.00 p.m. = 480 min of the first day + 08.00 a.m. to 08.59 a.m. = 59 min of the following day), and 779 min of nighttime (06.00 p.m. to 06.59 a.m.). Total time evaluated per day and animal was 1,318 min. The averages of all 8 days per animal were used for statistical analysis.

### Laboratory analysis

Herbage samples were oven-dried at 60°C for 48 h at the Institute of Agriculture and Animal Science, Rampur, Nepal, weighed and ground to pass a sieve of 0.75 mm mesh size on a Thomas mill. In Switzerland samples were analyzed for proximate contents following standard procedures (AOAC 1997; Van Soest et al. 1991). Dry matter and total ash were assessed on an automatic thermogravimetric determinator (TGA-500, Leco, St.Joseph, MI, USA: AOAC index no. 942.05) and nitrogen with a C/N analyzer (Analysator CN-2000, Leco, St.Joseph, MI, USA; AOAC 977.02). Crude protein (CP) was calculated as 6.25 × N. Contents of neutral detergent fiber (NDF), acid detergent fiber and acid detergent lignin were assessed on a Fibertec System M (Tecator 1020 Hot Extraction, Höganäs, Sweden). The methods were based on Van Soest et al. (1991), data were corrected for ash content, and for NDF analysis α-amylase and sodium sulfite were used. Hemicellulose was calculated as neutral detergent fiber − acid detergent fiber, and cellulose was computed as acid detergent fiber − acid detergent lignin.

### Statistical analysis

The mixed procedure of SAS (version 9.3) was used to perform the analysis of variance. The botanical composition was analyzed with Model 1 treating pasture site (S) as single fixed effect and paddock as replicate $\left( {{\text{Y}}_{\text{ij}} = {\text{ S}}_{\text{i}} + \, \varepsilon_{\text{ij}} } \right)$. The plots used for biomass cutting (three/paddock) were averaged per paddock, and paddock was used as replicate resulting in *n* = 4 (Sites 1 and 5) and *n* = 6 (Sites 2–4).

For ECM yield, milk composition, BW and behavior data, a model was applied exclusively on data of the hybrids in order to determine site (S) and SD effects. This Model 2 reads:

$$ {\text{Y}}_{\text{ijk}} = {\text{ S}}_{\text{i}} + {\text{ SD}}_{\text{j}} + {\text{ S }} \times {\text{ SD}}_{\text{ij}} + \, \varepsilon_{\text{ijk}} $$

where S and SD as well as their interaction were treated as fixed effects. Site was treated as repeated variable, animal (replicate) nested within SD was set as subject and paddock as random effect. For milk data, the last 3 days were averaged.

Model 3 compared the two genotypes for BW data, ECM yield and milk composition at Sites 2–4 with low SD only. It reads as follows:

$$ {\text{Y}}_{\text{ijk}} = {\text{ S}}_{\text{i}} + {\text{ G}}_{\text{j}} + {\text{ S }} \times {\text{ G}}_{\text{ij}} + \, \varepsilon_{\text{ijk}} $$

with G (genotype) and S (site) and the interaction. Both S and G were treated as fixed effects, S was treated as repeated variable, and animal (replicate) nested within genotype was considered as subject. Paddock was set as a random effect. The data obtained on the last 3 days were averaged for milk data. Multiple comparisons among means were performed with Tukey’s method considering *P* < 0.05 as significant. The data on the chemical composition of the herbage selected by the experimental animals are arithmetic means. Means of variables subjected to analysis of variance and given in the tables are Least Square means.

## Results

### Sward biomass and functional groups, and chemical composition of the herbage selected

The biomass available was largest (*P* < 0.05) at Site 2 (Table 1). In the upward movement the pastures were still in earlier stages of growth, as characterized for instance by the prevalence of herbs at Site 1 (>90%, *P* < 0.05 compared to the other sites). Together, Poaceae and Cyperaceae contributed to >50% of forage biomass on Sites 2–5. Poaceae alone made up about half of the biomass at Site 5, whereas they accounted for <10% at Site 1. During the downward movement, the vegetation matured, was fully flowering at Site 4 and relatively dry with only some regrowth mostly by grasses underneath the senescent material at Site 5. Accordingly, the increasing maturity of the vegetation was associated with a decline in nutritional quality of the herbage selected. The CP content in the herbage selected was numerically highest at Site 1 and values were gradually lower when moving from Site 1 to Site 5. The relatively high CP content found at Site 1 could be related to the early sprouting stage in spring and in addition to the low prevalence of grass species. Cellulose and hemicellulose contents of the herbage selected increased numerically from Sites 1–5, but this pattern was not reflected in the lignin contents.

**Table 1:** Total biomass (air-dry matter) available and chemical composition of the forage selected per pasture site

Plant group	Pasture site					SEM	*P* value
	1	2	3	4	5		
Biomass (g/m^2^)	107^c^	231^a^	122^bc^	199^ab^	96^c^	20.2	<0.001
Plant groups^A^							
Poaceae (%)	6^b^	34^a^	31^a^	36^a^	50^a^	5.5	<0.001
Cyperaceae (%)	<1^b^	22^a^	27^a^	24^a^	16^ab^	3.7	<0.001
Herbs (%)	92^a^	43^b^	39^b^	40^b^	34^b^	4.6	<0.001
Chemical composition of the forage selected (g/kg dry matter)							
Organic matter	881	813	916	902	917	–	–
Crude protein	260	218	190	196	91	–	–
Neutral detergent fiber	452	550	581	597	690	–	–
Acid detergent fiber^B^	312	373	338	363	423	–	–
Acid detergent lignin^B^	94	139	87	106	85	–	–
Hemicellulose	140	177	243	234	268	–	–
Cellulose	218	234	251	257	338	–	–

Means within a row carrying different superscripts differ at *P* < 0.05.

Recorded before starting the respective experimental measurements (Sites 1 and 5: 12 cuttings in *n* = 4 paddocks; Sites 2–4: 18 cuttings in *n* = 6 paddocks).

Values are means of samples pooled over 7 days of sampling with two samples per paddock (A, B), resulting in *n* = 8 samples for Sites 1 and 5 and *n* = 12 samples for Sites 2–4.

*SEM* standard error of the mean.

^A^Remainder to 100% are’others’ (ferns, small shrubs, mosses) which are not shown.

^B^A and B samples per paddock and site were pooled before analysis resulting in *n* = 2.

### Performance of hybrids and yaks

There were various effects of pasture site, SD and genotype in the traits describing performance of the experimental animals (Table 2). In the hybrids, only pasture site had an effect (*P* < 0.001) on initial BW, while BW remained unaffected (*P* > 0.1) by SD. There was daily BW gain in the hybrids at Sites 1–2 and at Site 4, whereas there was a BW loss at Sites 3 and 5. The ECM yield was affected by both, pasture site and SD (*P* < 0.001). At high compared to low SD, a significant reduction in ECM yield occurred at Sites 2 and 3. However, ECM yield/unit of pasture area available per animal was significantly higher with high SD on Sites 1–3, but not on Sites 4–5. Site, but not SD, had an effect on the contents of fat, protein and lactose in the milk of the hybrids. On average, the hybrids were heavier (*P* < 0.001) than the yaks. There was a significant interaction between genotype and pasture site in body weight change. While the hybrids lost 653 g BW/day/animal at the highest altitude at Site 3, which was different (*P* < 0.05) from the BW gain of 897 g/day/animal at Site 2, the yaks gained 836 g BW/day (*P* > 0.05 compared to 728 g/day/animal at Site 2, data not shown in table). Comparing the initial BW of yaks and hybrids measured at Sites 2 and 4, the hybrids gained 26 kg across this time, while the yaks increased their BW by 49 kg. Looking specifically at the high altitude Site 3, the hybrids numerically lost on average 2.3% of their BW across the 9 days of measurement period at low SD, whereas the yaks gained on average 3.6% of BW. An interaction of pasture site and genotype occurred with an initially higher (*P* < 0.05) ECM yield in the hybrids at Site 2 (3.97 vs. 3.11 kg/day/animal (*P* < 0.05) in hybrids and yaks at Site 2, respectively, data not shown in table). There was an interaction of genotype and pasture site in milk fat content and fat amount. No significant differences in milk fat content were found in the hybrids when comparing Sites 2 and 4 (5.88 and 6.62%, respectively), but the yaks had a higher (*P* < 0.05) milk fat content at Site 4 compared to Site 2 (7.07 and 5.26%, respectively). Yaks had higher (*P* < 0.05) milk protein and lactose contents than the hybrids.

**Table 2:** Performance of hybrids (low and high SD, *n* = 4 and *n* = 8, respectively) and in comparison to yaks (Sites 2–4, low SD, n = 4)

Traits	SD	Pasture site (S) and stocking density (SD) effects (hybrids only)^A^									Site (S) and Genotype (G) effect (low SD only)					
		Site					SEM	*P*-values			Genotype		SEM	*P*-values		
		1	2	3	4	5		S	SD	S × SD	Hybrids^B^	Yak^C^		G	S	G × S
Initial body weight (kg)	Low	201^de^	229^abc^	255^ab^	255^ab^	262^ab^	7.7	<0.001	0.98	0.59	247	210	6.1	<0.001	<0.001	0.25
	High	202^ce^	229^bd^	261^a^	259^a^	251^ab^										
Body weight change (g/day/animal)	Low	1,290^a^	897^a^	−653^b^	347^ab^	−497^b^	238.6	<0.001	0.73	0.62	197	588	220.5	0.23	0.014	<0.001
	High	673^a^	834^a^	−447^b^	458^ab^	−451^b^										
Energy-corrected milk (kg/day/animal)	Low	3.55^ab^	3.99^a^	3.04^b^	2.94^bcd^	1.93^efg^	0.180	<0.001	<0.001	0.13	3.30	3.16	0.05	0.08	0.023	0.006
	High	3.02^bc^	2.73^bce^	2.40^cde^	2.07^df^	1.23^g^										
Pasture efficiency (g ECM/day and m^2^)^D^	Low	4.13^bcd^	2.68^d^	2.70^d^	2.80^bcd^	2.75^bcd^	0.335	<0.001	<0.001	0.014	2.71	2.66	0.04	0.32	0.003	0.012
	High	7.00^a^	3.68^c^	4.27^b^	3.93^bcd^	3.50^bcd^										
Fat (g/day/animal)	Low	153^abc^	182^a^	139^b^	144^bcd^	99^ef^	8.7	<0.001	<0.001	0.10	155	142	3.90	0.037	0.14	0.002
	High	131^bcde^	125^bcde^	112^cde^	103^de^	62^f^										
Protein (g/day/animal)	Low	119^ab^	123^a^	92^bcd^	80^cde^	45^fg^	5.7	<0.001	<0.001	0.22	98	107	3.58	0.08	<0.001	0.08
	High	101^abc^	84^cd^	71^def^	55^ef^	30^g^										
Fat (%)	Low	5.31^de^	5.88^bcde^	5.81^cde^	6.62^ab^	7.12^a^	0.163	<0.001	0.24	0.20	6.10	6.25	0.17	0.55	<0.001	0.019
	High	5.48^e^	5.99^bcd^	6.12^bc^	7.06^a^	7.05^a^										
Protein (%)	Low	4.16^ab^	3.92^abc^	3.85^abc^	3.65^bcd^	3.26^de^	0.120	<0.001	0.56	1.00	3.81	4.68	0.05	<0.001	0.21	0.047
	High	4.22^a^	3.98^ab^	3.90^ab^	3.75^bd^	3.38^ce^										
Lactose (%)	Low	4.95^ab^	4.92^bcd^	4.92^bcd^	4.90^cd^	4.92^bcd^	0.008	<0.001	0.38	0.69	4.92	4.98	0.01	<0.001	0.29	0.24
	High	4.97^a^	4.93^bcd^	4.92^bcd^	4.91^d^	4.93^bc^										

*SEM* standard error of the mean.

^A^Means of the same variable for high and low SD across pasture sites carrying different superscripts differ at *P* < 0.05.

^B^Only values from low stocking density and only from Sites 2–4 (S2–S4) were used. Morning and evening milk was used for analysis.

^C^As yaks were accompanied by their calves, the weigh-suckle-weigh method was applied for evaluating the milk yield (see “Methods”). Only morning milk was available for analysis.

^D^Calculated as ECM yield divided by m^2^/head of the respective SD per pasture site.

### Activity pattern of the hybrids

During the time the hybrids stayed at altitudes between 4,000 and 4,500 m, the number of steps per day declined (*P* < 0.05) from Sites 2–3 (Table 3). However, the size of the paddocks at Site 2 was larger than at Site 3 and especially Site 4, thus possibly affecting the number of steps. Walking time per day declined and lying time increased from Sites 2–4 (*P* < 0.05), whereas standing time did not change (*P* > 0.1). Overall, high SD resulted in more steps (*P* < 0.05) during the entire day and daytime. SD had overall an effect on lying and standing time (*P* < 0.05).

**Table 3:** Activity pattern of the hybrids kept at low and high SD on Sites 2–4

Period	Activity	SD	Site (S)^A^			SEM	*P*-values		
			2	3	4		S	SD	S × SD
Entire day	Number of steps	Low	6,188^ab^	4,458^c^	3,730^c^	289.1	<0.001	0.01	0.74
		High	7,085^a^	4,963^bc^	4,189^c^				
	Walking (min)	Low	434^ab^	362^bc^	289^c^	17.8	<0.001	0.04	0.81
		High	467^a^	369^bc^	307^c^				
	Standing (min)	Low	483^ab^	491^a^	461^ab^	20.1	0.73	<0.001	0.67
		High	400^b^	421^ab^	418^ab^				
	Lying (min)	Low	400^c^	464^bc^	568^a^	14.5	<0.001	<0.001	0.49
		High	451^c^	528^ab^	593^a^				
Daytime^B^	Number of steps	Low	3,402^ab^	3,023^b^	2,937^b^	233.5	0.01	0.02	0.38
		High	4,240^a^	3,691^ab^	3,168^b^				
	Walking (min)	Low	267^ab^	249^ab^	228^b^	15.8	0.02	0.08	0.71
		High	304^a^	275^ab^	237^ab^				
	Standing (min)	Low	220^ab^	230^ab^	282^a^	12.5	<0.001	<0.001	0.44
		High	183^b^	184^b^	273^a^				
	Lying (min)	Low	51^ab^	60^ab^	29^b^	9.5	<0.001	0.56	0.28
		High	52^ab^	79^a^	29^b^				

Means by treatment, *n* = 4 each for high and low SD.

*SEM* standard error of the mean.

^A^Means of the same variable for high and low SD across pasture sites carrying different superscripts differ at *P* < 0.05.

^B^Daytime = from morning milking to evening milking plus next day’s daylight hours before morning milking (480 + 59 = 539 min, respectively).

## Discussion

### Effects of stocking density on performance and activity pattern in cattle × yak hybrids

Pasture allowance or diet quality or both (Wales et al. 1999; Bovolenta et al. 2008), and SD (O’Donovan and Delaby 2008), have been repeatedly reported to influence MY in cattle. A higher SD typically decreases yields of milk and milk constituents (O’Brien et al. 1999), which is consistent with the present results. The average ECM yield decline of 26% with high SD is a sign that the limit to overstocking might have been approached. Still, the overall milk production per unit of pasture area was about 50% higher with the high SD as compared with the low SD. Although there was overall an effect of SD on ECM yield and yield of milk constituents, no effect of SD on initial BW or BW change was registered. Thus, in the present study using low-performing genotypes no partitioning towards maintaining milk supply to the calves at cost of BW was found, different from what has been described for higher yielding breeds kept on mountainous pastures (Bovolenta et al. 2008). The activity pattern of the hybrids was partly responding to SD. Lying time is considered as an indicator of comfort behavior (Gibbons et al. 2012). A study with sheep and goats on pasture (Animut et al. 2005) showed decreased lying times and increased standing times with increasing SD. In the Inner Mongolia, Lin et al. (2011) found in sheep an increase of grazing time during daytime hours with higher SD at cost of standing time but not of lying time. In the present study, grazing could not be separated from walking or standing (defined as ≤3 steps/min). However, consistent with Lin et al. (2011), the hybrids spent overall less time standing, and performed more steps at high compared to low SD. This may reflect a higher grazing competition inside the paddocks at high compared to low SD.

### Effects of pasture site on performance and activity pattern in cattle × yak hybrids

There were various effects of pasture site on ECM yield and milk composition. Pasture site effects within a transhumant cycle on different performance traits or milk FA profile were also found elsewhere (Leiber et al. 2006; De Noni and Battelli 2008; Gorlier et al. 2012). For their interpretation it is important to keep in mind that the factor ‘pasture site’ inseparably included individual effects of forage quality, amount of forage biomass on offer, altitude, the progressing stage of lactation and interactions among these factors. Generally, MY is impaired with increasing altitude, as has been reported for instance by Qiao et al. (2013) for Chinese Holstein cattle kept at 3,600 m a.s.l. as compared to 1600 m a.s.l. In the present study the decline in ECM yield was primarily caused by the well-known effect of the advancing stage of lactation during transhumance (e.g. Gorlier et al. 2012). In addition, energy supply and ECM yield were depressed by the decline of forage quality due to the progressing season. Consistent with Leiber et al. (2006) and, numerically, Gorlier et al. (2012), the milk fat content increased with altitude, but in the present study only when moving the animals from 3,200 to 4,000 m a.s.l. During the downward movement, milk fat content further increased due to the progressing stage of lactation (Ostersen et al. 1997) and the increasing fiber content of the forage. As expected, milk protein declined with increasing altitude (Leiber et al. 2006) and with low forage quality (Rook and Line 1961). Grazing at both, the highest altitude Site 3 (4,500 m) and Site 5, obviously resulted in an energy deficiency in the hybrids as indicated by BW changes, the first likely because of hypoxia (Bianca 1976) and the last due to the low forage quality. The BW gain found on Site 4 compared to Site 3 might indicate that this altitude was more comfortable for the hybrids whereas their high altitude tolerance was surpassed at 4,500 m. Fully adapted animals staying most of the time at very high altitude such as the yaks in the present study do not show such BW losses (Bartl et al. 2009; Qiao et al. 2013).

There was a pasture site effect in most of the activity parameters measured. However, the results of the pasture site effect, other than those of the SD effect, have to be interpreted with caution, as especially the number of steps taken might have been rather related to the different paddock sizes (largest at Site 2) than to the actual site conditions.

### Effects of genotype on performance

Yaks are especially well adapted to high altitude environments (Wiener et al. 2003). He et al. (2011) reported a clearly higher MY in yak × yellow cattle hybrids as compared to yaks, although the hybrids in that study were more advanced in lactation than the yaks (121 ± 29 vs 90 ± 30 days in milk, respectively). Different from the expectations, in the present experiment the hybrid type tested (Dimjo) was overall not clearly superior to yaks in ECM yield although the hybrids initially (at Site 2) performed better. This initial difference in ECM yield (kg/day) resembles the difference found by Barsila et al. (2014) in ECM yield for yaks (2.78) and Dimjos (3.48) when grazing at 4,700 m. Likewise, the hybrids had calved several weeks earlier than the yaks suggesting that the potential MY of the hybrids might have been higher when compared with yaks at the same stage of lactation. However, that the initial difference leveled out from Site 3 (highest altitude) onwards, together with the BW loss in the hybrids and BW gain in the yaks at Site 3, were indications for the better adaptation of yaks compared to the hybrids to very high altitudes.

## Conclusion

As hypothesized, overall a high stocking density limited the hybrid individuals’ milk yield even during a short period of 9 days. In the present study, SD had recurrent effects on milk yield as in the time between the experimental periods the animals were allowed to graze freely, and forced animals to perform overall more steps, likely to gather enough feed. However, the individual milk yield loss was compensated by a concomitantly about 50% higher overall milk yield per m^2^ of pasture area. The performance (energy-corrected milk yield) of the hybrids investigated was not superior to that of the yaks at the same low stocking density at very high altitude. It seems that the hybrids had surpassed their comfort zone at this altitude. However, long-term studies over several years are needed to be able to draw definitive conclusions.

## References

[CR2] Agroscope (2015) Fütterungsempfehlungen und Nährwerttabellen für Wiederkäuer (Feeding recommendations and nutrient tables for ruminants). http://www.agroscope.admin.ch/futtermitteldatenbank/04834/index.html?lang=de. Accessed 4 Ap 2015

[CR1] Aharoni Y, Henkin Z, Ezra A, Dolev A, Shabtay A, Orlov A (2009). Grazing behavior and energy costs of activity: a comparison between two types of cattle. J Anim Sci.

[CR3] Animut G, Goetsch AL, Aiken GE, Puchala R, Detweiler G, Krehbiel CR (2005). Grazing behavior and energy expenditure by sheep and goats co-grazing grass/forb pastures at three stocking rates. Small Rumin Res.

[CR4] AOAC (1997). Official methods of analysis.

[CR5] Barsila SR, Kreuzer M, Devkota NR, Ding L, Marquardt S (2014). Adaptation to Himalayan high altitude pasture sites by yaks and different types of hybrids of yaks with cattle. Livest Sci.

[CR6] Bartl K, Gómez CA, Aufdermauer T, Garcia M, Kreuzer M, Hess HD (2009). Effect of diet type on performance and metabolic traits of Peruvian local and introduced cow types kept at 200 and 3,600 m of altitude. Livest Sci.

[CR7] Bauer JJ (1990). The analysis of plant-herbivore interactions between ungulates and vegetation on alpine grasslands in the Himalayan region of Nepal. Vegetatio.

[CR8] Berry NR, Jewell PL, Sutter F, Edwards PJ, Kreuzer M (2002). Selection, intake and excretion of nutrients by Scottish Highland suckler beef cows and calves, and Brown Swiss dairy cows in contrasting Alpine grazing systems. J Agri Sci.

[CR9] Bianca W (1976). The significance of meteorology in animal production. Int J Biometeorol.

[CR10] Bovolenta S, Saccà E, Corazzin M, Gasperi F, Biasioli F, Ventura W (2008). Effects of stocking density and supplement level on milk production and cheese characteristics in Brown cows grazing on mountain pasture. J Dairy Res.

[CR11] Council For International Organizations Of Medical Sciences and The International Council For Laboratory Animal Science (2012) International Guiding Principles for Biomedical Research Involving Animals. http://grants.nih.gov/grants/olaw/Guiding_Principles_2012.pdf. Accessed 4 Apr 2015

[CR12] De Noni I, Battelli G (2008). Terpenes and fatty acid profiles of milk fat and ‘‘Bitto” cheese as affected by transhumance of cows on different mountain pastures. Food Chem.

[CR13] Dong SK, Wen L, Zhu L, Lassoie JP, Yan ZL, Shrestha KK (2009). Indigenous yak and yak-cattle crossbred management in the high altitude areas of northern Nepal: a case study from Rasuwa district. Afr J Agric Res.

[CR14] Gibbons J, Medrano-Galarza C, de Passillé AM, Rushen J (2012). Lying laterality and the effect of IceTag data loggers on lying behavior of dairy cows. Appl Anim Behav Sci.

[CR15] Gorlier A, Lonati M, Renna M, Lussiana C, Lombardi G, Battaglini LM (2012). Changes in pasture and cow milk compositions during a summer transhumance in the western Italian Alps. J Appl Bot Food Qual.

[CR16] He S, Ma Y, Wang J, Li Q, Yang X, Tang S (2011). Milk fat chemical composition of yak breeds in China. J Food Compos Anal.

[CR17] Joshi DD (1982). Yak and Chauri husbandry in Nepal.

[CR18] Leiber F, Kreuzer M, Leuenberger H, Wettstein H-R (2006). Contribution of diet type and pasture conditions to the influence of high altitude grazing on intake, performance and composition and renneting properties of the milk of cows. Anim Res.

[CR19] Lin L, Dickhoefer U, Müller K, Wurina, Susenbeth A (2011). Grazing behavior of sheep at different stocking rates in the Inner Mongolian steppe, China. Appl Anim Behav Sci.

[CR20] Mishra C, Prins HHT, van Wieren SE (2001). Overstocking in the trans-Himalayan rangelands of India. Environ Conserv.

[CR21] O’Brien B, Dillon P, Murphy JJ, Mehra RK, Guinee TP, Connolly JF (1999). Effects of stocking density and concentrate supplementation of grazing dairy cows on milk production, composition and processing characteristics. J Dairy Res.

[CR22] O’Donovan M, Delaby L (2008). Sward characteristics, grass dry matter intake and milk production performance is affected by timing of spring grazing and subsequent stocking rate. Livest Sci.

[CR23] Ostersen S, Foldager J, Hermansen JE (1997). Effects of stage of lactation, milk protein genotype and body condition at calving on protein composition and renneting properties of bovine milk. J Dairy Res.

[CR24] Pande TN, Yamamoto H (2006). Cattle treading effects on plant growth and soil stability in the mountain grassland of Japan. Land Degrad Develop.

[CR25] Qi XB, Jianlin H, Wang G, Rege JEO, Hanotte O (2009). Assessment of cattle genetic introgression into domestic yak populations using mitochondrial and microsatellite DNA marker. Anim Gen.

[CR26] Qiao GH, Shao T, Yu CQ, Wang XL, Yang X, Zhu XQ (2013). A comparative study at two different altitudes with two dietary nutrition levels on rumen fermentation and energy metabolism in Chinese Holstein cows. J Anim Physiol Anim Nutr.

[CR27] Rook JAF, Line C (1961). The effect of the plane of energy nutrition of the cow on the secretion in milk of the constituents of the solids-non-fat fraction and on the concentrations of certain blood-plasma constituents. Br J Nutr.

[CR28] Van Soest PJ, Robertson JB, Lewis BA (1991). Methods for dietary fiber, neutral detergent fiber, and nonstarch polysaccharides in relation to animal nutrition. J Dairy Sci.

[CR29] Wales WJ, Doyle PT, Stockdale CR, Dellow DW (1999). Effects of variations in herbage mass, allowance, and level of supplement on nutrient intake and milk production of dairy cows in spring and summer. Aust J Exp Agric.

[CR30] Wang N, Vandepitte W, Wu JC (1994). Crossbreeding yak (*Bos grunniens*) and yellow cattle (*Bos taurus*): simulation of a rotational system and estimation of crossbreeding means and heterosis effects. J Appl Anim Res.

[CR31] Wiener G, Jianlin H, Long, RJ (eds) (2003) The Yak, 2nd edn. RAP Publication 2003/06. FAO, Regional Office for Asia and the Pacific, Bangkok, Thailand

